# Targeting USP2 induces degradation of PML-RARα with or without drug-resistant mutations in acute promyelocytic leukemia: USP2 overcomes APL resistance via PML-RARα degradation

**DOI:** 10.3724/abbs.2025135

**Published:** 2025-08-12

**Authors:** Jie Zhang, Wenxuan Wu, Yun Wang, Youping Zhang, Yingying Wang, Wenhui Bai, Zhenge Zhang, Chujiao Zhu, Yunzhao Wu, Ziwei Zhang, Li Yang, Hu Lei, Hanzhang Xu, Li Zhou, Yingli Wu

**Affiliations:** 1 Shanghai Institute of Hematology State Key Laboratory of Medical Genomics National Research Center for Translational Medicine at Shanghai Ruijin Hospital Affiliated to Shanghai Jiao Tong University School of Medicine Shanghai 200025 China; 2 Hongqiao International Institute of Medicine Shanghai Tongren Hospital/Faculty of Basic Medicine Chemical Biology Division of Shanghai Universities E-Institutes Key Laboratory of Cell Differentiation and Apoptosis of the Chinese Ministry of Education Shanghai Jiao Tong University School of Medicine Shanghai 200025 China; 3 Shanghai Ruijin Rehabilitation Hospital Shanghai 200023 China; 4 Yusuf Hamied Department of Chemistry University of Cambridge Cambridge CB2 1EW UK

**Keywords:** acute promyelocytic leukemia, PML-RARα, USP2, ML364, drug resistance

## Abstract

Despite the high efficacy of all-trans retinoic acid (ATRA) and arsenic trioxide (ATO) in treating acute promyelocytic leukemia (APL), approximately 10%–20% of patients develop drug resistance due to mutations in PML-RARα and other factors. Here, we find that inhibition of USP2 with ML364 or USP2 silencing reduces PML-RARα protein levels in both ATRA-sensitive and ATRA-resistant APL cells, and this effect is reversed by proteasome inhibition. Conversely, USP2 overexpression enhances PML-RARα stability. Mechanistically, USP2 interacts with and deubiquitinates PML-RARα, including its drug-resistant mutants. Consistent with PML-RARα degradation, ML364 treatment significantly induces apoptosis in APL cell lines and primary leukemia cells. In conclusion, this study identifies USP2 as a novel deubiquitinating enzyme for PML-RARα and highlights USP2 inhibition as a potential therapeutic strategy for APL with PML-RARα mutations.

## Introduction

PML-RARα is generated by the t(15;17)(q24;q21) chromosomal translocation [1]. The resulting oncoprotein blocks cell differentiation and suppresses apoptosis, ultimately leading to the development of acute promyelocytic leukemia (APL) [ 2, 3] . The introduction of all-trans retinoic acid (ATRA) and arsenic trioxide (ATO) has significantly improved the overall survival of APL patients [ 1, 4, 5] . However, approximately 10%–20% of APL patients develop resistance to treatment with ATRA and/or ATO [6]. Among the known resistance mechanisms, the most prevalent one involve mutations in the PML-RARα fusion gene, particularly in the ligand-binding domain (LBD) of RARα and the PML-B2 domain, the primary target site of ATO [ 7– 9] . Mutations in the PML-B2 domain ( *e.g.*, A216V and L218P) may impair ATO therapeutic efficacy by disrupting proper ATO binding, interfering with post-translational modifications (such as SUMOylation and multimerization), and causing aberrant subcellular localization [ 10– 12] . These defects collectively lead to insufficient degradation and pathological retention of oncoproteins, ultimately sustaining their stability and driving therapeutic resistance by evading ATO-mediated clearance mechanisms. When mutations occur in the LBD of RARα, conformational changes can weaken its binding affinity to ATRA. Owing to this reduced binding capacity, the LBD may fail to properly dissociate nuclear receptor corepressor complexes and recruit activator complexes, leading to aberrant transcriptional regulation and subsequent ATRA resistance [ 13– 15] . One promising strategy to overcome this mutation-induced drug resistance is to induce protein degradation via alternative pathways. For example, targeting HDAC3 has been shown to induce the degradation of PML-RARα in ATRA- and ATO-resistant APLs [16].

Deubiquitinases (DUBs) regulate protein stability and function by removing ubiquitin chains from substrate proteins, modifying ubiquitin linkages, and processing ubiquitin precursors [17]. The stability of PML-RARα can also be influenced by DUB activity, suggesting that inhibiting specific DUBs may induce PML-RARα degradation and overcome ATRA- and ATO-resistance in patients with PML-RARα mutations. For example, the inhibition of YOD1, a member of the ovarian tumor protease (OTU) family, has been shown to promote PML-RARα degradation and effectively eliminate APL cells, including drug-resistant subtypes [18]. This work suggests that DUB inhibition is a potential therapeutic strategy for degrading PML-RARα.

In the present study, we identified USP2 as a novel DUB for PML-RARα. *USP2* knockdown or the use of the USP2 inhibitor ML364 induced the degradation of PML-RARα and the apoptosis of APL cells. Importantly, inhibition of USP2 could also degrade drug-resistant PML-RARα mutants. Our study demonstrated that USP2 is a novel target for the treatment of APL, particularly in patients with drug-resistant APL driven by PML-RARα mutations.

## Materials and Methods

### Cells and culture

Human embryonic kidney (HEK293T) cells were purchased from the American Type Culture Collection (ATCC; Manassas, USA). NB4 and MR2 cells were provided by Prof. Michel Lanotte (Hôpital Saint-Louis, Paris, France). NB4 and MR2 cells were cultured in RPMI-1640 medium (BasalMedia, Shanghai, China). HEK293T cells were cultured in Dulbecco’s modified Eagle’s medium (DMEM; BasalMedia). All of the media were supplemented with 10% fetal bovine serum (FBS; Vazyme, Nanjing, China) and 1% penicillin/streptomycin (BasalMedia). All the cell lines were maintained at 37°C in a humidified atmosphere containing 5% CO _2_. Primary APL blasts extracted from the bone marrow of APL patients (Ruijin Hospital, Shanghai Jiaotong University School of Medicine, Shanghai, China) were isolated via Lymphoprep™ (STEMCELL, Vancouver, Canada). Written informed consent from patients and approval from the Institutional Review Board of Ruijin Hospital, Shanghai Jiao Tong University School of Medicine (2021/154) were obtained before the use of these clinical materials for research purposes.

### Plasmids, reagents and antibodies

The PML-RARα plasmid was a kind gift from Prof. Kan-Kan Wang (Shanghai Institute of Hematology, Ruijin Hospital, Shanghai Jiao Tong University School of Medicine). PML-RARα drug-resistant mutants (A216V, L218P, R276Q, and ΔF286) were kind gifts from Prof. Jiong Hu (Ruijin Hospital, Shanghai Jiao Tong University School of Medicine). The PLZF-RARα plasmid was preserved in our laboratory. USP2 ^WT^ (USP2 wild-type), USP2 ^C276S^ (USP2 catalytic mutant), USP2 (1–258 amino acids) and USP2 (259–605 amino acids) plasmids were constructed in our laboratory. The HA-USP8 plasmid was purchased from YouBio (Changsha, China). The USP2 shRNAs were purchased from Tsingke Biotechnology (Shanghai, China). The USP2 shRNA-1 and USP2 shRNA-2 sequences are as follows: USP2 shRNA #1, 5′-GCTACACAGATGCCCACTATG-3′; and USP2 shRNA #2, 5′-GGAGTTCCTTCGCTTTCTTCT-3′.

ML364, b-AP15, degrasyn and the USP25/28 inhibitor AZ1 were purchased from MedChemExpress (Monmouth Junction, USA). P22077 was purchased from EMD Millipore (Burlington, USA). ATRA, ATO, MG132, CQ, and cycloheximide (CHX) were purchased from Sigma-Aldrich (St. Louis, USA).

Antibodies against RARα (#62294) were purchased from Cell Signaling Technology (Danvers, USA). Antibodies against PML (sc-71910) were purchased from Santa Cruz (Dallas, USA). Antibodies against USP2 (10392-1-AP) and β-actin (66009-1-Ig) were purchased from Proteintech (Wuhan, China). Antibodies against Myc (AE070), HA (AE105), and Flag (AE063) were purchased from ABclonal (Wuhan, China). Antibodies against His (ab18184) were purchased from Abcam (Shanghai, China).

### Lentivirus production and transduction

HEK293T cells were cotransfected with the transfer plasmid and packaging plasmids (psPAX2 and pMD2.g). After transfection for 6 h, the medium was replaced by fresh pre-warmed complete medium. After another 48 h, the viral particles were collected, resuspended in RPMI-1640 medium and then frozen at –80°C. NB4 and MR2 cells were infected with the virus in the presence of polybrene (8 μg/mL) to increase the infection efficiency. After 3 days of infection, the cells were screened with puromycin (1 μg/mL) for at least 5 days.

### Immunoprecipitation and western blotting

For immunoprecipitation assays, the cells were lysed in lysis buffer (20 mM Tris-HCl, pH 7.5; 150 mM NaCl; 0.1 mM EDTA; 0.2% Triton X-100) supplemented with protease inhibitor cocktail (MedChemExpress) on ice for 30 min. After centrifugation at 13,000 *g* for 15 min at 4°C, the supernatants were incubated with primary antibodies at 4°C overnight. The next day, protein A/G agarose beads (Beyotime, Shanghai, China) were added and incubated for 5–6 h. Next, the beads were washed with washing buffer (20 mM Tris-HCl, pH 7.5; 150 mM NaCl; 0.1 mM EDTA; 1% Triton X-100) three times. Finally, the bound proteins were dissolved in 5× SDS-PAGE sample loading buffer and analyzed via western blotting. For ubiquitylation analysis, MG132 (15 μM) was added 4–6 h before cell collection.

After the corresponding treatment, the cells were collected and lysed with 2× SDS sample loading buffer. Protein extracts were electrophoresed via 6%–10% SDS-PAGE gels (Meilunbio ^®^, Dalian, China) and transferred to nitrocellulose membranes (Bio-Rad, Hercules, USA). The membranes were blocked with 5% non-fat milk at room temperature for 1 h and incubated with primary antibodies overnight at 4°C. The membranes were then incubated with a horseradish peroxidase (HRP)-conjugated secondary IgG antibody at room temperature for 1 h. The membranes were finally imaged via chemiluminescence (ECL, Amersham, UK).

### Cell proliferation and cell viability assay

To assess cell viability, the cells were collected and counted via a Countstar automated cell counter. Before counting, trypan blue was used to distinguish between viable and dead cells.

A cell proliferation assay was performed via a Cell Counting Kit-8 (Meilunbio ^®^). The cells were cultured in 96-well or 384-well plates and then treated with appropriate concentrations of drugs. After incubation for 2–3 days, CCK-8 solution was added to each well and incubated for 2–4 h. Finally, the optical density (OD) at 450 nm was detected via a microplate reader.

### Cell apoptosis and differentiation analysis

Cell apoptosis was detected via Annexin V and 7-aminoactinomycin D (7-AAD) or propidium iodide (PI) staining. After treatment, the cells were collected, washed with PBS and then stained according to the manufacturer’s protocol. Early apoptotic cells (Annexin V-positive, 7-AAD- or PI-negative) and late apoptotic cells (Annexin V-positive, 7-AAD- or PI-positive) were then detected with a flow cytometer (BD Biosciences, San Diego, USA). The results were analyzed via CytExpert 2.4 software. The kits used included the 488-Annexin V and PI Apoptosis Kit (Green), the 647A-Annexin V and PI Apoptosis Kit (Red) (SB-Y6002, SB-Y6026; ShareBio, Shanghai, China) and the Annexin V-APC/7-AAD Apoptosis Kit (AP105; Liankebio, Hangzhou, China).

Cell differentiation was evaluated by detecting the expression levels of cell surface markers of differentiation. After being treated with ATRA and ML364, the cells were collected, washed with PBS and then stained with a FITC-labeled anti-CD11b antibody (ab128797; Abcam) according to the manufacturer’s protocol. The number of differentiated cells (CD11b positive) was then determined with a flow cytometery (BD Biosciences). The results were analyzed via CytExpert 2.4 software.

### CHX chase assay

Cells were cultured in 6-well plates at appropriate densities and treated with 10 μg/mL CHX, with experimental groups receiving 6 μM ML364 and control groups receiving an equivalent volume of DMSO. Following 0, 4, 8, 12, and 24 h of treatment, cells were collected, lysed, and analyzed via western blotting to determine target protein expression levels.

### Immunofluorescence assay

The cells were first fixed with 4% paraformaldehyde for 10 min and then permeabilized and blocked with QuickBlock™ blocking buffer (P0260; Beyotime) for immunofluorescence staining for 10 min. The cells were subsequently incubated with the anti-PML antibody (dilution 1:50) and anti-USP2 antibody (dilution 1:100) overnight at 4°C, followed by incubation with the HRP-conjugated secondary antibody (dilution 1:200) for 2 h at room temperature. The cell nuclei were stained with 4,6-diamidino-2-phenylindole (DAPI, C1002; Beyotime) before being observed with a microscope. Confocal imaging was performed via a laser confocal microscope (Nikon, Nagoya, Japan).

### Proximity ligation assay (PLA)

A Duolink® *in situ* proximity ligation assay was used to detect the interactions between USP2 and PML-RARα. NB4 cells were prepared as above for immunofluorescence staining before being incubated overnight at 4°C with primary antibodies against PML (dilution 1:50) and USP2 (dilution 1:100). After washing, PLUS and MINUS PLA probes (DUO92004 and DUO92002; Sigma-Aldrich) were hybridized at 37°C for 1 h. Hybridized probes were ligated with the Ligation-Ligase system at 37°C for 30 min, and then amplified using the Amplification-Polymerase system (DUO92008; Sigma-Aldrich) at 37°C for 100 min. After sequential washes with Wash Buffers A and B (DUO82049; Sigma-Aldrich), slides were finally mounted with Duolink® In Situ Mounting Medium with DAPI (DUO82040; Sigma-Aldrich).

### Statistical analysis

The data were obtained from three independent experiments and are expressed as the mean ± standard deviation (SD). The statistical significance of differences between groups was determined by unpaired two-tailed Student’s *t* test and one-way analysis of variance (ANOVA). All the data were analyzed via GraphPad Prism 8 (GraphPad software, La Jolla, USA). The results were considered significant when *P*  < 0.05.

## Results

### ML364 triggers PML-RARα degradation and shows a strong inhibitory effect on APL cells

**Figure 1:**
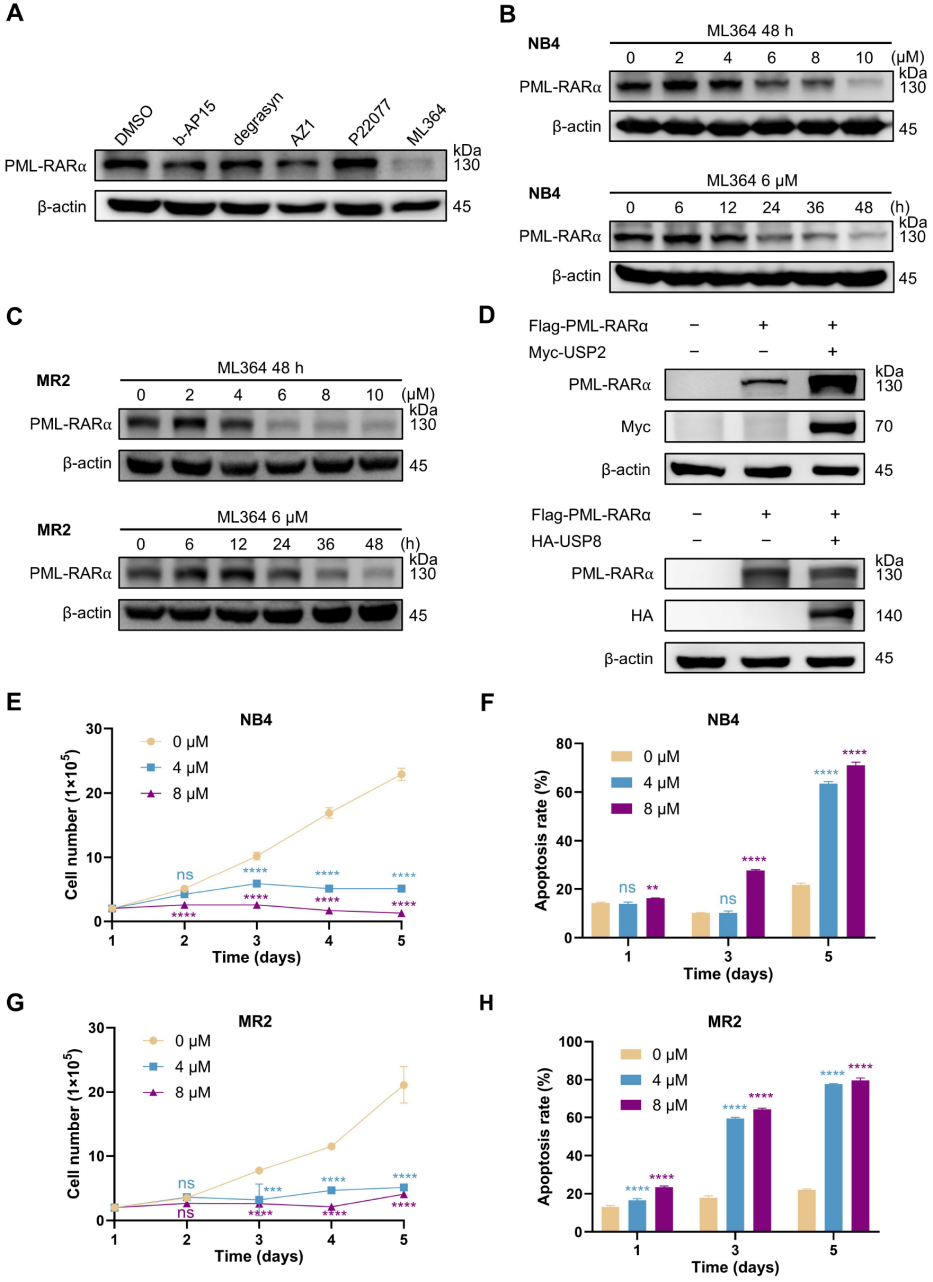
Figure 1 ML364 triggers PML/RARα degradation and strongly inhibits APL cells (A) HEK293T cells stably expressing PML-RARα were treated with 5 DUB inhibitors at the respective IC50 concentrations for 24 h, and the indicated proteins were assessed by western blotting. All the western blot bands labeled PML-RARα in this study were immunoblotted with an anti-RARα antibody. (B) NB4 cells were exposed to various concentrations of ML364 for 48 h (top) or to 6 μM ML364 for the indicated time intervals (bottom). (C) MR2 cells were exposed to various concentrations of ML364 for 48 h (top) or to 6 μM ML364 for the indicated time intervals (bottom). (D) HEK293T cells were cotransfected with Flag-PML-RARα and Myc-USP2 or HA-USP8 plasmids, and the levels of exogenous PML-RARα were determined via western blotting. (E,G) The proliferation of NB4 and MR2 cells exposed to the specified concentrations of ML364 was assessed via the trypan blue exclusion test at the indicated time points. (F,H) The apoptosis rates of NB4 and MR2 cells exposed to the specified concentrations of ML364 were assessed via flow cytometry analysis at the indicated time points. Annexin V-positive cells were quantified with CytExpert software. (E–H) Data are presented as the mean ± SD (n = 3); *P < 0.05, **P < 0.01, ***P < 0.001, and ****P < 0.0001 vs 0 μM. The significance analysis was conducted via one-way ANOVA.

To identify potential deubiquitinating enzymes that regulate the stability of PML-RARα, we treated NB4 cells with five reported DUB inhibitors ( Supplementary Table S1) and examined the protein levels of PML-RARα. Among these compounds, ML364 significantly reduced the PML-RARα protein level ( Figure 1A). To further validate this finding, we treated NB4 cells with various doses of ML364 for different time intervals. The results demonstrated that ML364 decreased the protein level of PML-RARα in a dose- and time-dependent manner ( Figure 1B). Similar phenomena were observed in ATRA-resistant MR2 cells ( Figure 1C and Supplementary Figure S1).

ML364 has been previously reported as an inhibitor of USP2 and USP8 [19]. To determine which USP regulates PML-RARα expression, we overexpressed USP2 or USP8 in HEK293T cells transfected with exogenous PML-RARα ( Figure 1D). Interestingly, the accumulation of PML-RARα protein was observed only in cells overexpressing USP2. These data suggest that targeting USP2 with ML364 can reduce PML-RARα protein level.

Given the importance of PML-RARα for the survival of APL cells and the ability of ML364 to promote PML-RARα degradation by inhibiting USP2, we evaluated whether ML364 could inhibit APL cell proliferation and alleviate drug resistance. Treatment of NB4 cells with ML364 effectively suppressed their proliferation and induced their apoptosis ( Figure 1E,F). Similarly, ML364 significantly reduced the proliferative capacity of MR2 cells ( Figure 1G) and induced a substantial amount of apoptosis in these cells ( Figure 1H). However, ML364 treatment did not alter the expression of CD11b, indicating that it did not induce differentiation in NB4 or MR2 cells ( Supplementary Figure S2). These results suggest that ML364 may reduce the protein level of PML-RARα and inhibit the growth of APL cells by targeting USP2.

### *USP2* knockdown decreases the protein level of PML-RARα and leads to effective APL elimination

**Figure 2:**
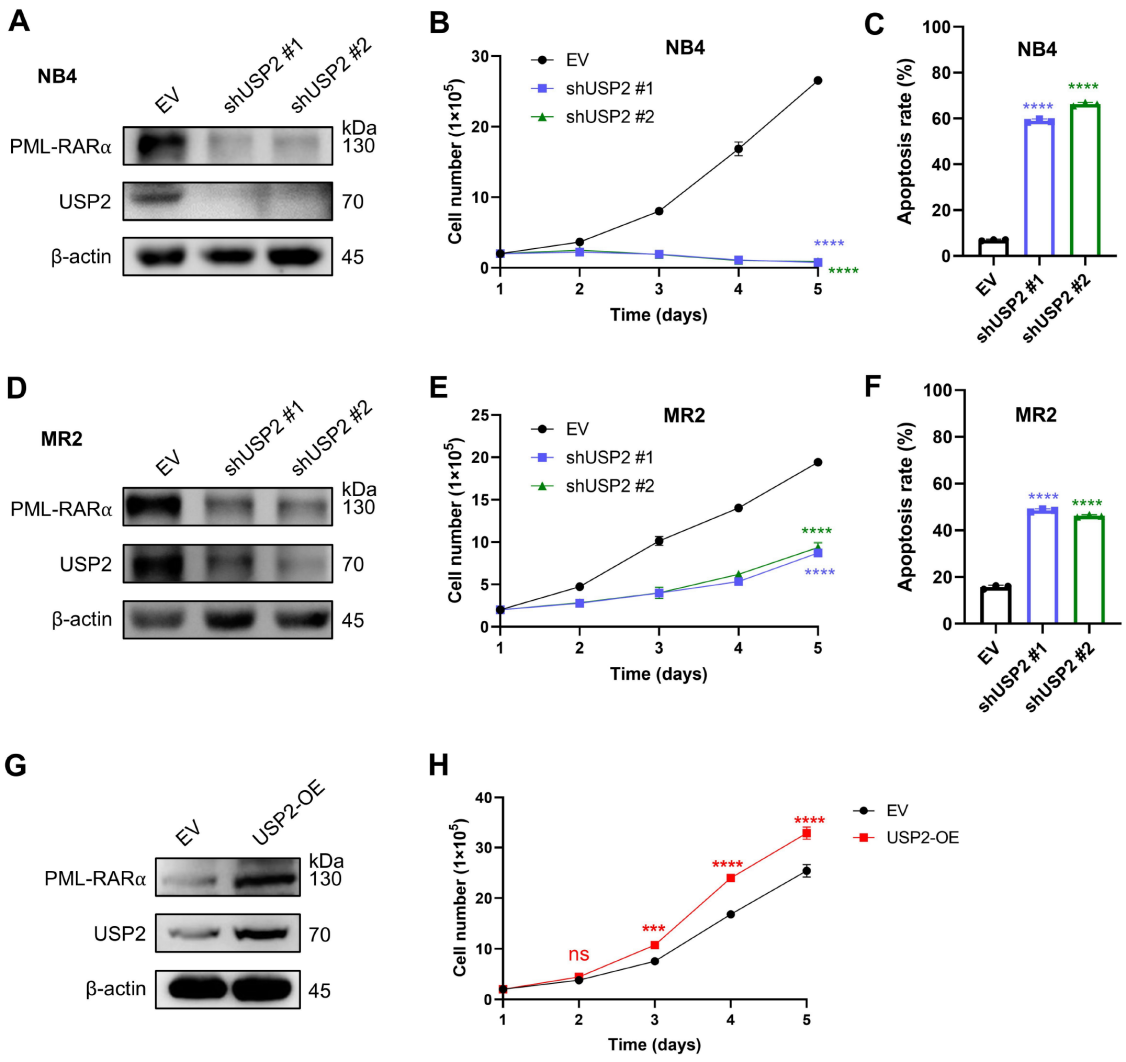
Figure 2 *USP2* knockdown decreases the protein level of PML-RARα and leads to effective APL elimination USP2 expression was downregulated with shUSP2 #1 or shUSP2 #2 in NB4 cells. (A) The levels of the indicated proteins were measured via western blotting. (B) The proliferation of NB4 cells was assessed via the trypan blue exclusion test at the indicated time points. (C)The percentage of apoptotic cells was assessed via flow cytometry analysis. Annexin V-positive cells were quantified with CytExpert software. USP2 was knocked down by shUSP2 #1 and shUSP2 #2 in MR2 cells. (D) The levels of the indicated proteins were measured via western blotting. (E) The proliferation of MR2 cells was assessed via the trypan blue exclusion test at the indicated time points. (F) The percentage of apoptotic cells was assessed via flow cytometry analysis at the indicated time points. Annexin V-positive cells were quantified with CytExpert software. USP2 was overexpressed in NB4 cells. (G) The levels of the indicated proteins were measured via western blotting. (H) The proliferation of NB4 cells overexpressing USP2 was assessed via the trypan blue exclusion test at the indicated time points. Data are presented as the means ± SD (n = 3); *P < 0.05, **P < 0.01, ***P < 0.001, and ****P < 0.0001 vs EV. The significance analysis was conducted via one-way ANOVA.

To further elucidate the role of USP2 in acute promyelocytic leukemia (APL), we knocked down *USP2* in NB4 cells and evaluated its effects on the PML-RARα protein level, cell proliferation, and cell death. Consistent with previous findings, *USP2* knockdown led to a significant decrease in PML-RARα protein level ( Figure 2A). Compared with the control, *USP2* knockdown markedly inhibited NB4 cell proliferation ( Figure 2B) and induced significant apoptosis ( Figure 2C and Supplementary Figure S3A). Similarly, *USP2* knockdown in MR2 cells resulted in decreased PML-RARα protein level ( Figure 2D), inhibited proliferation ( Figure 2E), and promoted apoptosis ( Figure 2F and Supplementary Figure S3B). Conversely, overexpression of USP2 increased PML-RARα protein level ( Figure 2G) and enhanced NB4 cell proliferation ( Figure 2H). These results collectively confirm that USP2 plays a crucial role in maintaining PML-RARα protein stability and promoting the proliferation of APL cells.

### USP2 regulates the stability of PML-RARα through the ubiquitin-proteasome pathway

**Figure 3:**
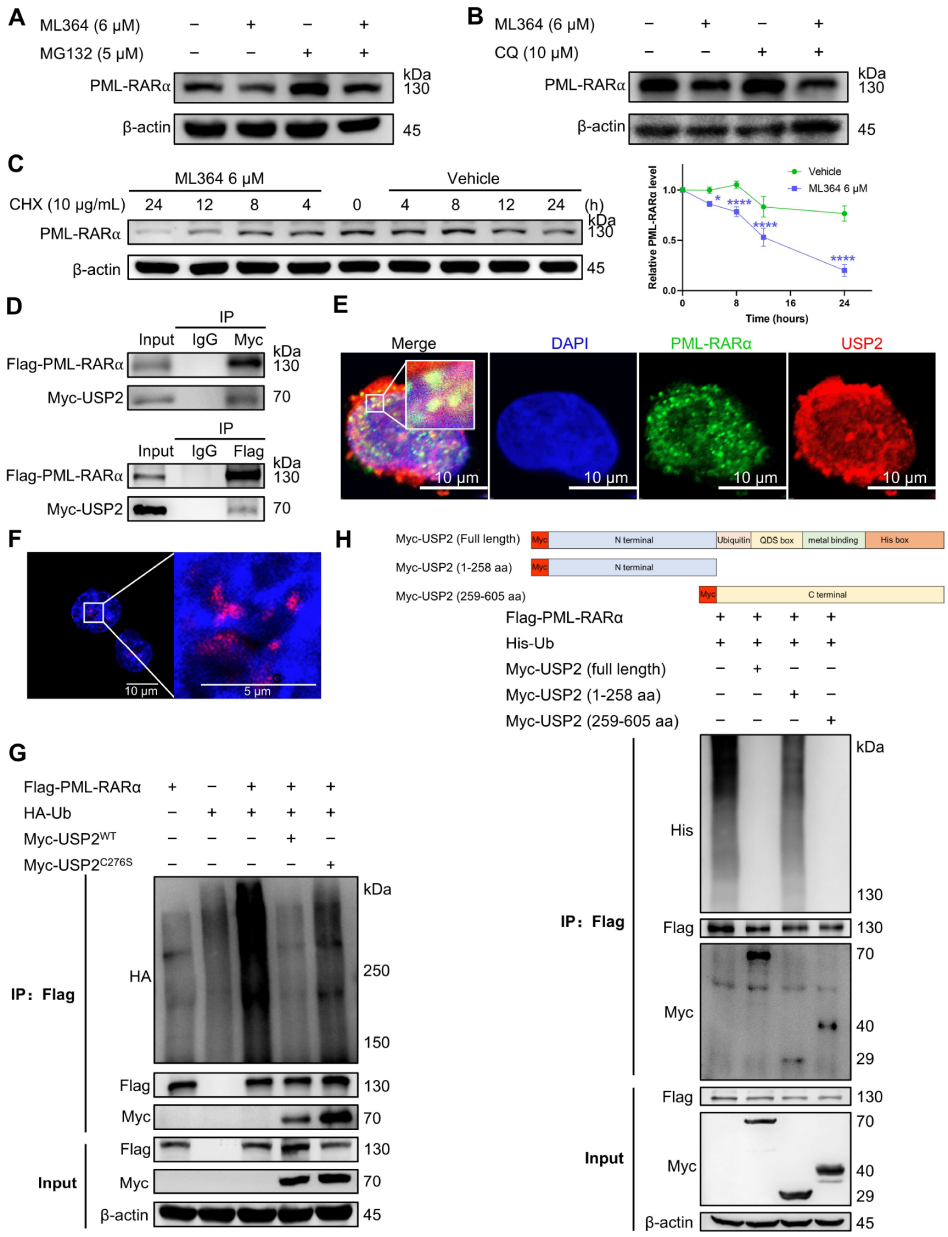
Figure 3 USP2 regulates the stability of PML-RARα through the ubiquitin-proteasome pathway (A) NB4 cells were treated with 6 μM ML364 in the presence or absence of 5 μM MG132 for 24 h. The protein levels were measured via western blotting. (B) NB4 cells were treated with 6 μM ML364 in the presence or absence of 10 μM CQ for 24 h. The protein levels were measured via western blotting. (C) Left: NB4 cells were treated with CHX (10 μg/mL) in the presence or absence of ML364 (6 μM) for the indicated time intervals. Right: Relative PML-RARα levels were analyzed via ImageJ. The data are presented as the mean ± SD (n = 3); *P < 0.05, **P < 0.01, ***P < 0.001, and ****P < 0.0001 vs Vehicle. The significance analysis was conducted via one-way ANOVA. (D) HEK293T cells were cotransfected with Flag-PML-RARα and Myc-USP2. The cell lysates were immunoprecipitated and immunoblotted with antibodies against Flag and Myc. (E) Colocalization of PML-RARα and USP2 was detected by immunostaining in NB4 cells. Scale bar: 10 μm. (F) Interactions between PML-RARα and USP2 were detected by PLA in NB4 cells. Blue: nuclei, red: PLA signals. Scale bars: 5 μm and 10 μm. (G) The deubiquitinating effect of USP2 on PML-RARα in cells. HEK293T cells were cotransfected with Flag-PML-RARα, His-Ub, Myc-USP2WT, and Myc-USP2C276S as indicated. The cells were treated with MG132 (15 μM) for 6 h before being collected and then immunoprecipitated with an anti-Flag antibody. Ubiquitination of PML-RARα was detected via western blotting with an anti-HA antibody. (H) Top: truncations of USP2 with a Myc tag. Bottom: HEK293T cells were cotransfected with the indicated plasmids. The cells were treated with MG132 (15 μM) for 6 h before being collected and then immunoprecipitated with an anti-Flag antibody. Ubiquitination of PML-RARα was detected via western blotting with an anti-His antibody.

Given that ML364 inhibits the deubiquitinating activity of USP2, we hypothesized that the ML364-induced downregulation of PML-RARα might be mediated through the ubiquitin‒proteasome pathway. As expected, the proteasome inhibitor MG132, but not the autophagy inhibitor chloroquine (CQ), significantly prevented the decrease in PML-RARα level induced by ML364 treatment ( Figure 3A,B). Additionally, CHX chase assay results revealed that ML364 treatment accelerated PML-RARα degradation in NB4 cells ( Figure 3C). These data indicate that ML364 reduces the protein level of PML-RARα by increasing its proteasome-mediated degradation.

To further confirm that targeting USP2 could regulate the ubiquitin-mediated degradation of PML-RARα, we first performed co-immunoprecipitation assays to examine the physical interaction between USP2 and PML-RARα. The results revealed that Myc-USP2 interacts with Flag-PML-RARα ( Figure 3D). Immunofluorescence assays and PLA experiments also demonstrated that USP2 and PML-RARα colocalize in APL cells ( Figure 3E,F). Next, we investigated whether USP2 regulates the ubiquitination of PML-RARα. We transfected HEK293T cells with HA-Ub, Flag-PML-RARα, Myc-USP2 ^WT^, and its catalytic-site mutant, Myc-USP2 ^C276S^, and examined the ubiquitination of PML-RARα. The results revealed that the overexpression of USP2 ^WT^, but not USP2 ^C276S^, significantly reduced the ubiquitination level of PML-RARα ( Figure 3G).

To map the specific domain of USP2 responsible for interacting with and regulating the stability of PML-RARα, we truncated USP2 into USP2 (1–258 aa) and USP2 (259–605 aa) and performed immunoprecipitation and western blot analysis. The results indicated that the catalytic domain is crucial for USP2 to interact with and deubiquitinate PML-RARα ( Figure 3H).

### USP2 also stabilizes different drug-resistant PML-RARα mutants

**Figure 4:**
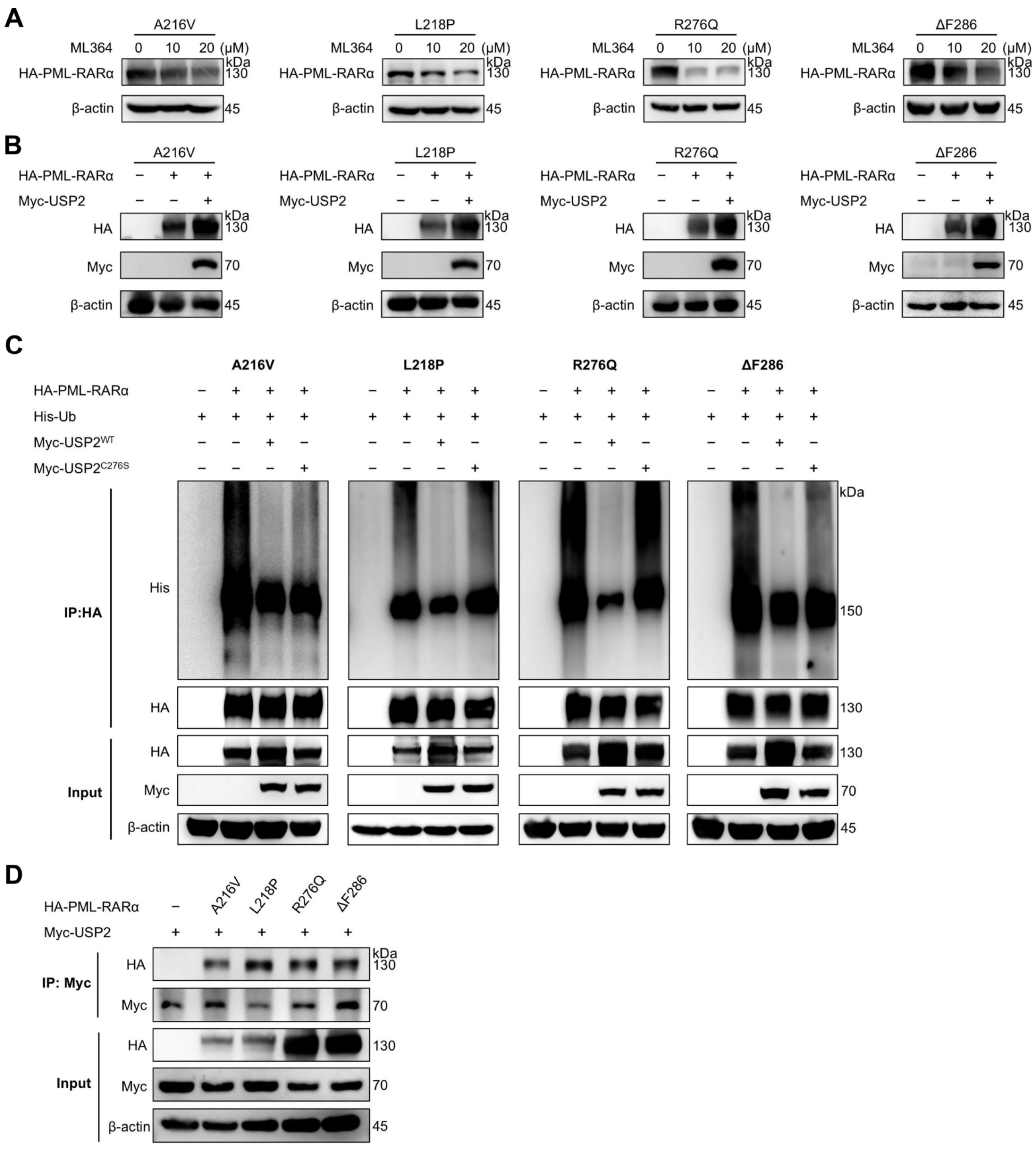
Figure 4 USP2 also stabilizes different drug-resistant PML-RARα mutants (A) HEK293T cells were transfected with the indicated plasmids for 24 h. Then, the cells were exposed to the indicated concentrations of ML364 for another 24 h. The cell lysates were immunoblotted with an anti-HA antibody. (B) HEK293T cells were cotransfected with Myc-USP2 and the indicated plasmids. The cell lysates were immunoblotted with an anti-HA antibody. (C) HEK293T cells were transfected with the indicated plasmids. The cells were treated with MG132 (15 μM) for 6 h before being collected and then immunoprecipitated with an anti-HA antibody. Ubiquitination of PML-RARα was detected via western blotting with an anti-His antibody. (D) The interactions between PML-RARα drug-resistant mutants (A216V, L218P, R276Q, and ΔF286) and USP2 were detected by immunoprecipitation. HEK293T cells were transfected with the indicated plasmids. The cell lysates were immunoprecipitated with an anti-Myc antibody and immunoblotted with an anti-HA antibody.

Clinical observations have shown that the ligand-binding domain (LBD) of RARα and the PML-B2 domain are prone to mutations that confer resistance to all-trans retinoic acid (ATRA) and arsenic trioxide (ATO). We hypothesized that USP2 might also regulate the stability of these mutant proteins. To test this hypothesis, we transfected drug-resistant mutants, including ATO-resistant mutants (A216V and L218P) and ATRA-resistant mutants (R276Q and ΔF286) ( Supplementary Figure S4), into HEK293T cells and treated them with varying concentrations of ML364. As shown in Figure 4A, ML364 effectively decreased the protein levels of these mutants.

To verify the role of USP2 in regulating the stability of PML-RARα mutants, we overexpressed USP2 alongside the aforementioned drug-resistant PML-RARα mutants. Interestingly, USP2 overexpression led to increased protein level of each of the four resistant PML-RARα mutants ( Figure 4B). Moreover, USP2 significantly reduced the ubiquitination levels of the resistant PML-RARα mutants, providing further evidence that USP2 may deubiquitinate PML-RARα ( Figure 4C).

In reciprocal immunoprecipitation assays, overexpression of HA-tagged drug-resistant PML-RARα was detected in the immunocomplexes of Myc-USP2 ( Figure 4D). These findings suggest that USP2 not only interacts with and regulates the stability of wild-type PML-RARα but also does so for mutant forms of PML-RARα.

### ML364 inhibits proliferation and promotes apoptosis of APL primary cells

**Figure 5:**
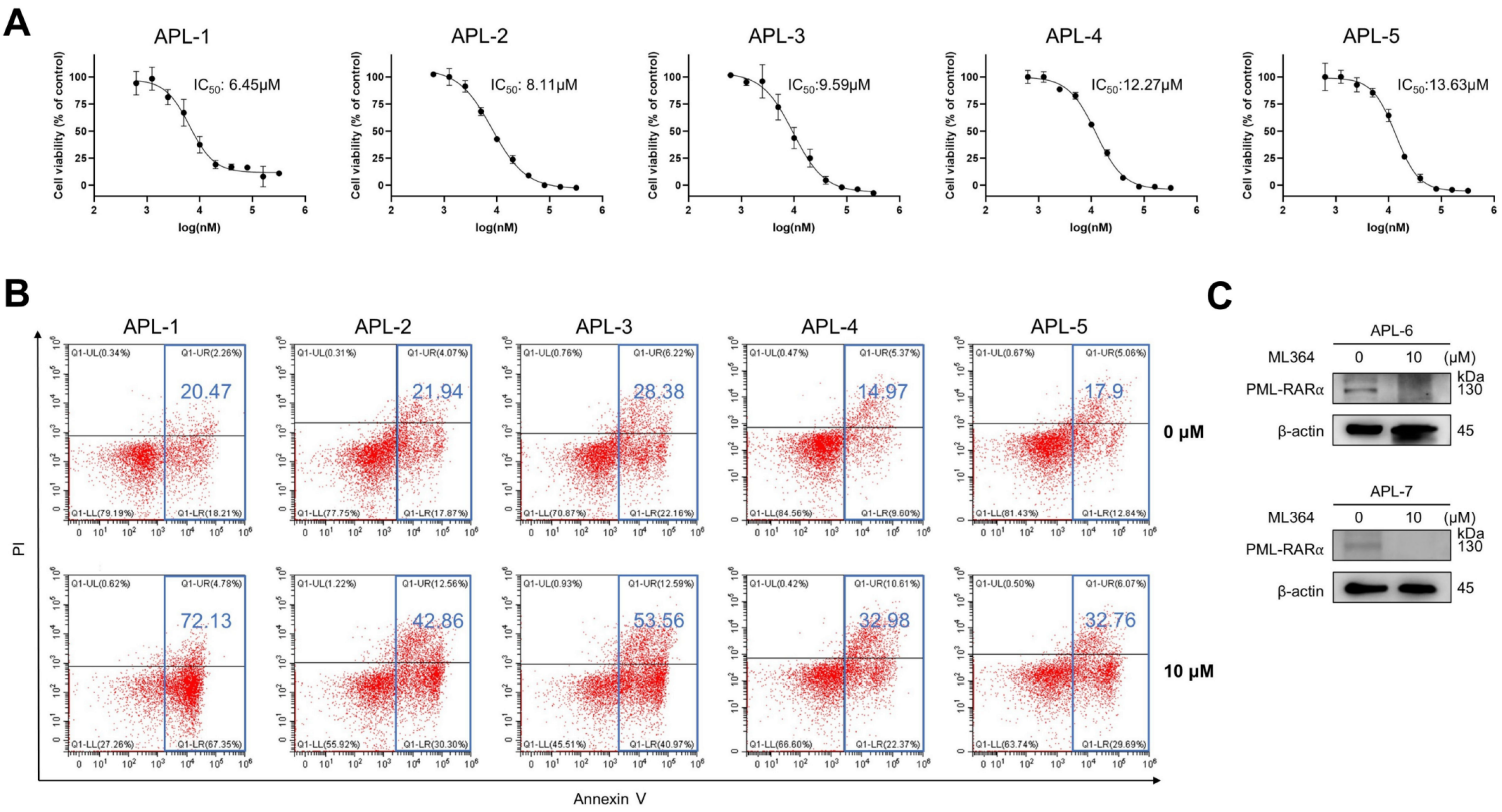
Figure 5 ML364 inhibits proliferation and promotes the apoptosis of APL primary cells (A) Primary APL cells derived from the bone marrow of 5 patients were treated with the indicated concentrations of ML364 for 72 h. CCK-8 assay was used to analyze the IC50 value of ML364 in primary APL cells. (B) The apoptosis rate of primary APL cells treated with or without ML364 (10 μM) for 72 h. Annexin V-positive cells were quantified with CytExpert software. (C) Primary APL cells were treated with 10 μM ML364 for 72 h. The protein levels were measured via western blotting.

To further validate the effectiveness of ML364, we treated primary APL blasts with this compound. ML364 significantly inhibited cell proliferation in a dose-dependent manner ( Figure 5A). After 72 h of treatment, the IC _50_ values of the five primary APL blasts ranged from 6.45 μM to 13.63 μM. ML364 also induced significant apoptosis in these cells, with the maximum apoptosis rate reaching 72.13% ( Figure 5B). Furthermore, the PML-RARα protein level was significantly reduced in primary APL cells after ML364 treatment ( Figure 5C). These results indicate that ML364 effectively inhibits the proliferation of primary APL cells.

## Discussion

Inducing the degradation of PML-RARα is a promising strategy to combat acute promyelocytic leukemia. In this study, we identified USP2 as a novel DUB for PML-RARα. USP2 stabilizes PML-RARα by removing its ubiquitin chains, thereby preventing its degradation. Inhibition of USP2 promotes the degradation of PML-RARα, including its drug-resistant mutants. Our findings suggest that USP2 is a potential therapeutic target for overcoming PML-RARα mutation-induced resistance to ATRA and ATO.

The combination of ATRA and ATO synergistically induces PML-RARα degradation, leading to long-term remission in APL patients. However, a subset of patients develop resistance due to mutations in PML or RARα, which hinder ATRA or ATO binding, preventing their therapeutic effects. Therefore, identifying novel strategies to degrade PML-RARα is crucial. PML-RARα degradation is mediated through the ubiquitin-proteasome system, where E3 ubiquitin ligases add ubiquitin and DUBs remove it. To promote PML-RARα degradation, we can either activate E3 ligases or inhibit DUBs. Since developing an E3 ligase activator is technically challenging, targeting DUBs with small-molecule inhibitors is a more practical approach. Identifying DUBs that regulate PML-RARα stability is therefore essential. Several DUBs, including YOD1 and USP37, have been reported to regulate PML-RARα or PLZF-RARα stability [ 18, 20] . In this study, we identified USP2 as a novel DUB for PML-RARα. Mechanistically, USP2 interacts with and removes ubiquitin from PML-RARα, leading to its stabilization. Conversely, USP2 inhibition promotes PML-RARα degradation. These findings confirm that USP2 is a bona fide DUB for PML-RARα.

A significant finding of this study is that USP2 regulates not only wild-type PML-RARα but also its drug-resistant mutants. These findings suggest that targeting USP2 could overcome ATRA and ATO resistance in APL patients. Interestingly, USP2 also regulates the stability of PLZF-RARα, another pathogenic fusion protein associated with ATRA and ATO resistance in APL ( Supplementary Figure S5A, B)[ 21, 22] . These findings highlight USP2 as a valuable therapeutic target for APL driven by RARα fusion proteins.

As a deubiquitinating enzyme, USP2 plays a broad role in cell proliferation, apoptosis, and metabolism [ 23– 25] . It is known to be involved in multiple solid tumors, including prostate cancer, liver cancer, colorectal cancer, and breast cancer [ 26– 28] . However, its role in hematological malignancies remains largely unexplored. Recently, we reported that USP2 is involved in myeloma by regulating KRAS stability [29]. In this study, we provide further evidence that USP2 is a promising target for APL therapy. The USP2 inhibitor ML364 significantly inhibited cell proliferation and induced apoptosis in both ATRA-sensitive and ATRA-resistant APL cells, as well as in primary APL cells. ML364 represents a promising candidate for treating APL. USP2 has been reported to regulate multiple substrates, including Cyclin D1, MDM2, and KRAS, which can independently influence cell proliferation and apoptosis [ 19, 29, 30] . Our study revealed that USP2 stabilizes PML-RARα and that its depletion reduces APL cell viability. However, we cannot exclude the possibility that the degradation of other substrates, such as Cyclin D1, may also contribute to the observed anti-leukemic effects.

In conclusion, we identified USP2 as a novel DUB that regulates the stability of PML-RARα and its drug-resistant mutants. Our findings suggest that targeting USP2 represents a potential therapeutic strategy for treating ATRA- and ATO-resistant APL.

## Supporting information

Supplementary_Information_25218

## Supplementary Data

Supplementary data are available at *Acta Biochimica et Biophysica Sinica* online.
