# Supplementary material for: Targeting USP2 induces degradation of PML-RARα with or without drug-resistant mutations in acute promyelocytic leukemia: USP2 overcomes APL resistance via PML-RARα degradation
Source: Acta Biochim Biophys Sin (Shanghai). 2025 Aug 12;58(3):574–83. doi: 10.3724/abbs.2025135 (PMC13059761; doi:10.3724/abbs.2025135)
Supplement: Supplementary_Information_25218 [file Supplementary_Information_25218.docx]

**Supplementary Table S1. The information and IC_50_ values of 5 DUB inhibitors**

| **No.** | **DUB inhibitor** | **CAS** | **Target** | **IC_50_ value (μM)** |
| --- | --- | --- | --- | --- |
| 1 | b-AP15 | 1009817-63-3 | USP14, UCHL5 | 1 |
| 2 | Degrasyn (WP1130) | 856243-80-6 | USP9x, USP5, USP14, UCH37 | 1 |
| 3 | USP25/28 inhibitor AZ1 | 2165322-94-9 | USP25/28 | 2 |
| 4 | P22077 | 1247819-59-5 | USP7, USP47 | 10 |
| 5 | ML364 | 1991986-30-1 | USP2, USP8 | 10 |

**­­**

**Supplementary figures.**


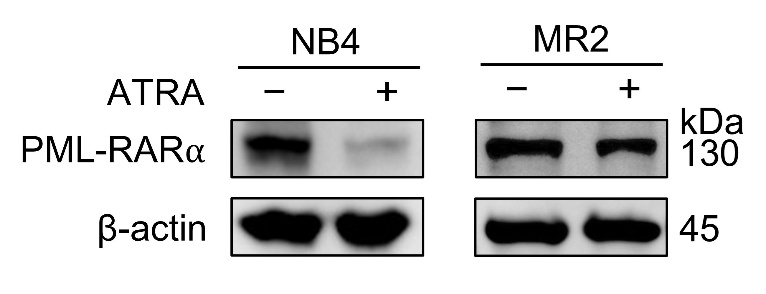


**Supplementary Figure S1. Verification of ATRA resistance** NB4 and MR2 cells were treated with 1 μM ATRA for 24 h and the indicated proteins were assessed by western blotting.


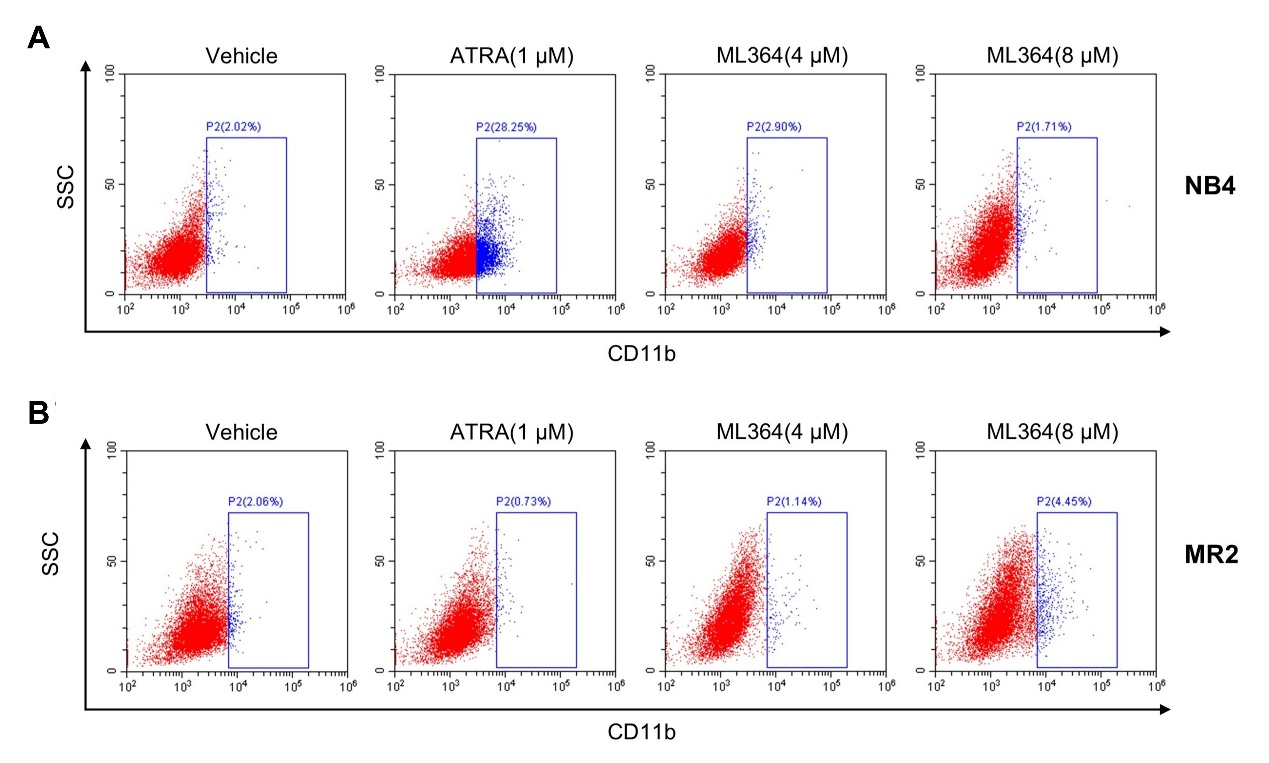


**Supplementary Figure S2. The effect of ML364 on cell differentiation** NB4 (A) and MR2 (B) cells were treated with ATRA and ML364 at the indicated concentrations for 72 h. Flow cytometry was used to analyze whether cell differentiation has been induced. CD11b-positive cells were quantified with CytExpert software.


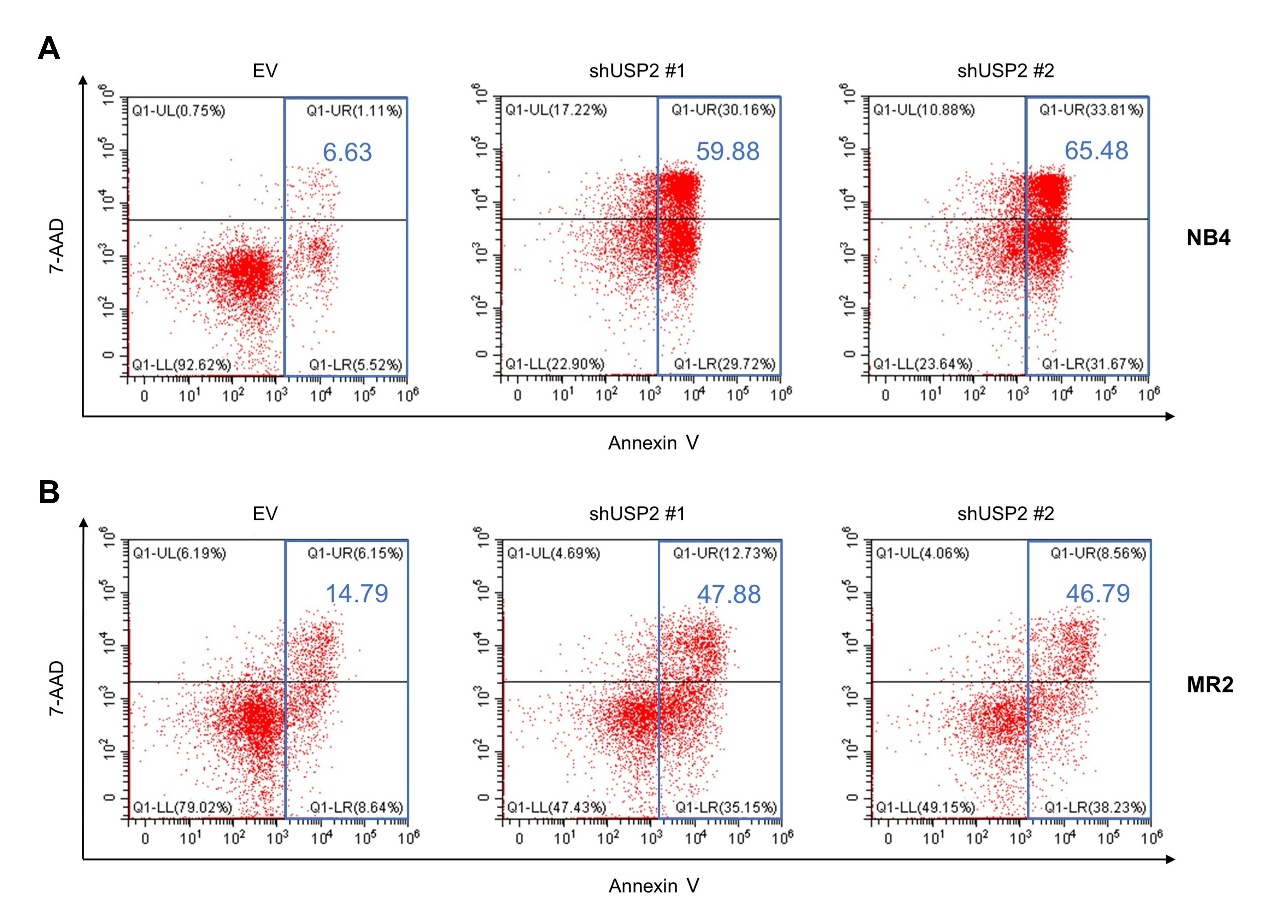


**Supplementary Figure S3. Knockdown of USP2 leads to apoptosis in APL cells** Knockdown of *USP2* was performed using shUSP2 #1 or shUSP2 #2. The apoptosis rate of NB4 (A) and MR2 (B) cells was assessed by flow cytometry analysis. Annexin V-positive cells were quantified with CytExpert software.


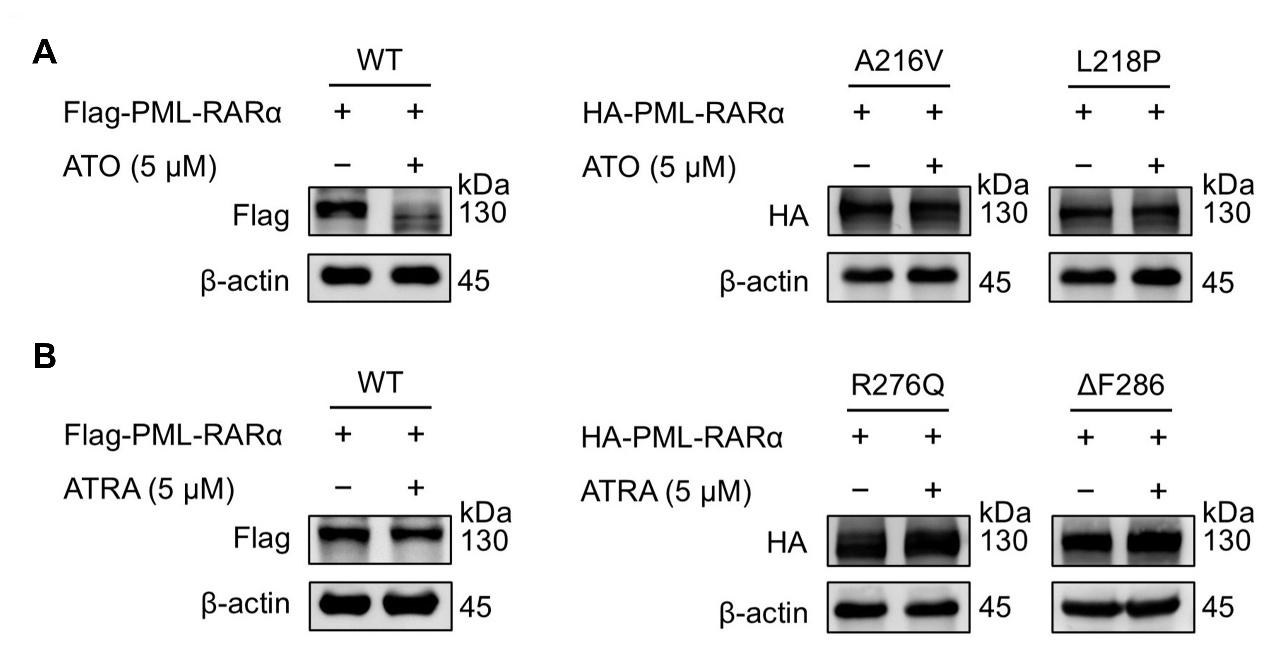


**Supplementary Figure S4.** **Validation of PML-RARα drug-resistant mutants (A216V, L218P, R276Q and ΔF286)** HEK293T cells were transfected with the indicated plasmids for 24 h. Then cells were exposed to ATO (A) or ATRA (B) for another 24 h. Cell lysates were immunoblotted with anti-Flag or anti-HA antibody.


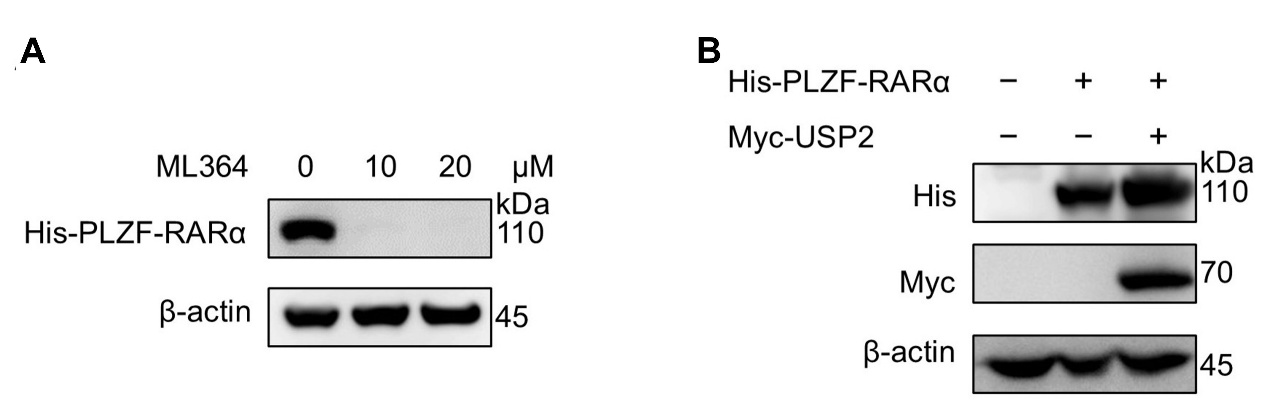


**Supplementary Figure S5. Effect of ML364 and USP2 overexpression on PLZF-RARα** (A) HEK293T cells were transfected with His-PLZF-RARα for 24 h. Then cells were exposed to the indicated concentrations of ML364 for another 24 h. Cell lysates were immunoblotted with anti-His antibody. (B) Effect of USP2 overexpression on the protein level of PLZF- RARα. HEK293T cells were transfected with Myc-USP2 and His-PLZF-RARα.
